# Theoretical Study on Electronic, Magnetic and Optical Properties of Non-Metal Atoms Adsorbed onto Germanium Carbide

**DOI:** 10.3390/nano12101712

**Published:** 2022-05-17

**Authors:** Lin Zhang, Zhen Cui

**Affiliations:** 1 School of Science, Xi’an University of Technology, Xi’an 710048, China; zhangliner@xaut.edu.cn; 2 School of Automation and Information Engineering, Xi’an University of Technology, Xi’an 710048, China

**Keywords:** non-metal, germanium carbide, absorption energy, work function, absorption spectrum

## Abstract

Nine kinds of non-metal atoms adsorbed into germanium carbide (NM-GeC) systems wereare investigated by first-principles calculations. The results show that the most stable adsorption positions vary with the NM atoms, and C-GeC exhibits the strongest adsorption. The adsorption of NM atoms causes changes in the electronic, optical and magnetic properties of the GeC system. F- and Cl-GeC turn into magnetic metals, P-GeC becomes a half-metal and H- and B-GeC appear as non-magnetic metals. Although C- and O-GeC remain non-magnetic semiconductors, N-GeC presents the behaviors of a magnetic semiconductor. Work function decreases in H-, B- and N-SiC, reaching a minimum of 3.37 eV in H-GeC, which is 78.9% of the pristine GeC. In the visible light region, redshifts occur in the absorption spectrum of C-GeC , with strong absorption in the wavelength range from 400 to 600 nm. Our analysis shows that the magnetism in semiconducting NM-GeC is attributed to the spinning state of the unbonded electrons of the NM atoms. Our study demonstrates the applications of NM-GeC in spintronics, optoelectronics and photovoltaic cells, and it provides a reference for analyzing magnetism in semiconducting NM materials.

## 1. Introduction

Graphene with a planar structure has attracted significant attention in academia and industries for its unique mechanical, electrical and thermal properties [1,2]. However, the zero-band gap and difficulties in integration with silicon-based systems hinder the application of graphene in electronics [3], which facilitates the exploration of other alternative two-dimensional (2D) atomic crystals [4,5,6]. An increasing number of new 2D materials have been investigated by first-principles calculations, including not only single-atom crystals [7,8], but also II–VI group compounds [9], III–V group compounds [10,11] and IV-IV group compounds [12,13,14]. These 2D materials have shown their advantages in the fields of spintronics [15,16], optoelectronics [17,18,19,20], energy conversion [21,22], catalysis [23,24,25,26] and gas sensing [27,28,29].

As the compounds of group IV elements, 2D silicon carbide (SiC) and germanium carbide (GeC) have stable planar honeycomb structures and exhibit similar properties to graphene [30,31]. Both of them are semiconductors whose band gaps are comparable to the energy of water oxidation and reduction [32,33]. Although 2D GeC exhibits excellent compatibility with silicon-based microelectronic devices [34], there are fewer studies on GeC than there are on SiC. To expand the applications of 2D GeC, impurity atoms are injected to modulate physical and chemical properties by doping or adsorption [35,36]. Previous studies on other 2D materials provide many effective analyses for property variations induced by dopants. For example, impurity atoms lead to the redistribution of charge and induce magnetism near the host atoms. The ferromagnetic states in transition metals (TM) atoms of adsorbed 2D materials are caused by the occupation mode of the *d* orbital hybridization of the TM atoms [37,38]. Magnetism in non-metal (NM) doped 2D blue phosphorene is related to the *sp*^3^ orbital hybridization of the NM atoms [39]. In addition, the interaction force between impurity atoms and neighboring atoms adjusts the bandgap of 2D materials and leads to the modulation of the absorption spectrum and emission electron capability [37].

Until now, some progress has been made in studying GeC systems. NM atoms adsorbed onto GeC (NM-GeC), such as 1H-GeC, have been predicted to be promising materials for digital circuits and light-emitting diodes [40]. Surface-functionalized GeC monolayers with F and Cl have demonstrated their advantage in opto-electronic devices for their strong absorption in the near ultraviolet light region [41]. The effective growth of GeC films has been achieved [42]. However, regularity conclusions on GeC systems are still lacking. In this work, nine kinds of NM-GeC structures are constructed based on optimum thermodynamics. The electrical, magnetic and optical properties of NM-GeC are discussed systematically. F-, Cl-, P- and N-GeC exhibit magnetism. H-GeC presents a stronger electron-emitting capacity than other NM-GeC systems. In the absorption spectrum of C-GeC, redshift occurs in the visible light region. These performances expand the applications of NM-GeC in spintronics, optoelectronics and photovoltaic cells. Discussions on the magnetism of NM-GeC provide a reference for other materials of adsorbed 2D NM atoms.

## 2. Computational Methods and Theoretics

**Figure 1:**
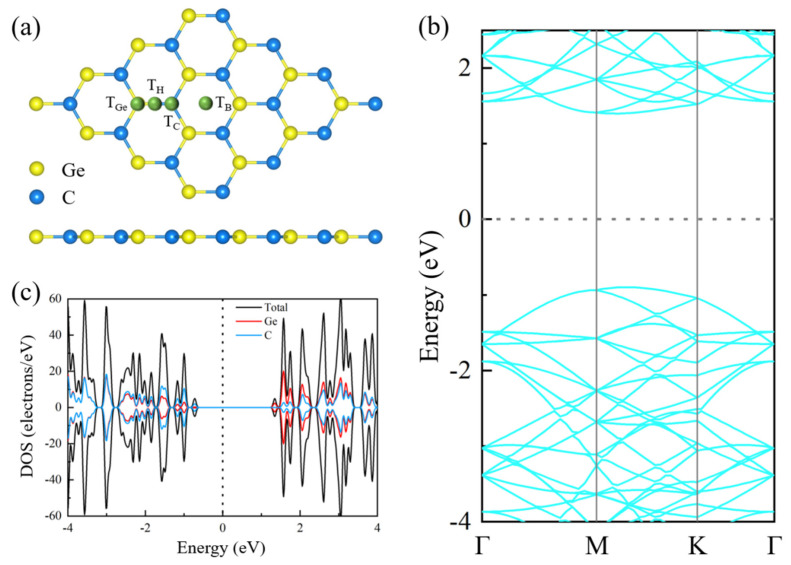
(**a**) The crystal structure, (**b**) the energy band structure and (**c**) the density of states (DOS) of pristine 2D GeC. T_C_ is above the C atom, T_Ge_ is above the Ge atom, T_H_ is above the Ge-C bond and T_B_ is above the center of the hexagonal structure. The spin-up component is above the Brillouin region, whereas the spin-down component is below the Brillouin region. The Fermi level is set to zero and is represented by a vertical black dotted line.

All first-principles calculations were performed with the Vienna Ab initio Simulation Package (VASP) based on density functional theory [43]. The parameterized exchange-correlation interactions were analyzed by the Generalized Gradient Approximation of the Perdew–Burke–Ernzerhof function [44,45,46]. A basis with a 550 eV cut-off energy of plane-waves was set to achieve high computational accuracy. Pristine 2D GeC was constructed with a 4 × 4 × 1 supercell configuration. To minimize the effect of the interaction between the adatoms, only one non-metal atom was injected, and the corresponding coverage concentration was 3.125%. All the possible adsorption positions were marked in the GeC supercell, as shown in Figure 1a. T_C_ was above the C atom, T_Ge_ was above the Ge atom, T_H_ was above the middle of the Ge-C bond and T_B_ was above the center of the hexagonal structure. A vacuum layer with a height of 20 Å was constructed along the vertical direction of the GeC plane to eliminate the interlayer interaction of periodic structures. The Brillouin zone was sampled by a set of 3 × 3 × 1 Monkhorst–Pack k-point grids [47]. All structures were fully relaxed until the Hellmann–Feynman force on each atom was less than 0.01 eV/Å, and the total energy change converged to 10^−5^ eV/atom or less. Local field effects were considered with random-phase approximation (RPA) [48].

The possibility of each NM-GeC structure was examined by calculating the corresponding adsorption energy,

(1) $$E_{\mathrm{ad}}=E_{\mathrm{NM}+\mathrm{GeC}}-(E_{\mathrm{GeC}}+E_{\mathrm{NM}})$$

where *E*_ad_ is the adsorption energy of the NM-SiC system. *E*_NM+GeC_, *E*_GeC_ and *E*_NM_ represent the energies of the final NM-GeC, the original isolated GeC and the isolated NM atom, respectively. A negative *E*_ad_ indicates that the adsorption process is thermodynamically favorable and that the NM-GeC system is more stable than the original system. However, the most likely structure corresponds to the system with the lowest adsorption energy. Therefore, the adsorption energies were compared at all the possible adsorption positions. The following discussions are all based on the most stable structures.

The adsorption of NM atoms caused the redistribution of charges, leading to changes in optical, electrical and magnetic properties. Charge density differences were introduced to display the charge transfer before and after the adsorption,

(2) $$\Delta \rho =(\rho_{\mathrm{GeC}}+\rho_{\mathrm{NM}})-\rho_{\mathrm{Total}}$$

where ∆*ρ* is the charge density difference (CDD) of the system. *ρ*_Total_, *ρ*_GeC_ and *ρ*_NM_ represent the charge densities of the non-metal atoms adsorbed onto GeC, of the pristine GeC, and of the isolated non-metal atom, respectively.

The redistribution of charge implies that new covalence bonds were formed between the adatom and the substrate atoms, which led to the change in the ability of the system to bind electrons. The work function was calculated to evaluate the electron-emitting capacity of NM-GeC,

(3) $$W=e\phi -E_{F}$$

where *W* is the work function, *e* is the charge of an electron, *ϕ* is the electrostatic potential in the vacuum near the surface and *E*_F_ is the Fermi level. The smaller that the work function is, the stronger that the electron-emitting capacity is.

As important optical parameter coefficients, the absorption coefficients of the pristine and NM-GeC were investigated by frequency-dependent dielectric response theory [49,50],

(4) $$\alpha (\omega )=\sqrt{2}\omega {\left[\frac{\sqrt{\varepsilon_{1}^{2}(\omega )+\varepsilon_{2}^{2}(\omega )}-\varepsilon_{1}(\omega )}{2}\right]}^{1/2}$$

where *ω* represents the photon frequency, and *ε*_1_(*ω*) and *ε*_2_(*ω*) are the real and imaginary parts of the complex dielectric function, respectively.

Spin-polarized charges were introduced to the analysis of the magnetic properties of the pristine GeC and NM-GeC,

(5) $$\rho =\rho_{\mathrm{up}}-\rho_{\mathrm{down}}$$

where *ρ* is the spin-polarized charge density (SPCD), and *ρ*_up_ and *ρ*_down_ represent the up and down-spin-polarized charge densities. When the electronic states of the spin-up and spin-down-polarized charges were asymmetric, the corresponding NM-GeC system exhibited magnetism.

## 3. Results and Discussion

The crystal structure of the pristine two-demensional GeC is depicted in Figure 1a. GeC is an indirect semiconductor with a bandgap of 2.3 eV. The conduction band minimum (CBM) and the valence band maximum (VBM) are located at points Γ and X, respectively, as shown in Figure 1b. According to the density of state (DOS) in Figure 1c, the Ge atom is the main contributor to the CBM of GeC, and the C atom is the main contributor to the valence band maximum (VBM). Considering the symmetry of the electronic states of spin-up and spin-down, the pristine GeC is non-magnetic.

**Figure 2:**
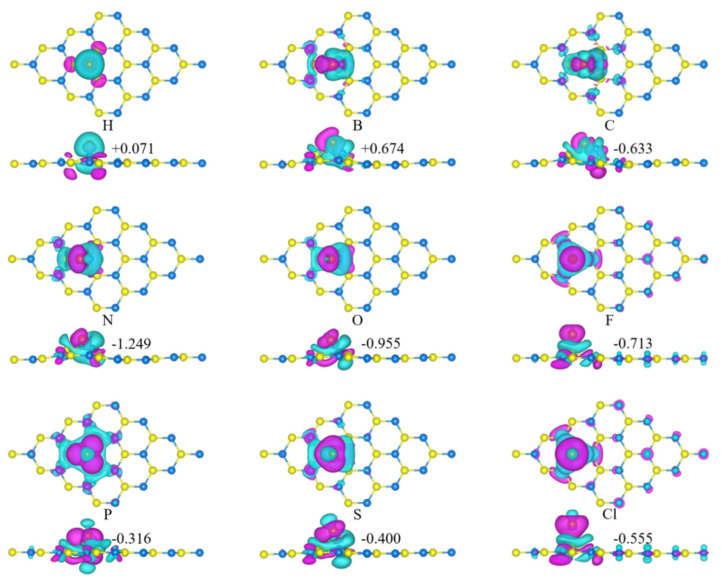
The charge density differences in the non-metal atoms adsorbed into GeC systems. Blue denotes the depletion of electrons, whereas purple represents the accumulation of electrons. The isovalue is set to 0.001 e/Å^3^. The blue, yellow and red balls are the C, Ge and NM atoms, respectively.

The adsorption energies of NM-GeC were compared at the four possible symmetric positions. The adsorption energies with the most stable structures are listed in Table 1, and their top and side views of the charge density difference (CDD) are depicted in Figure 2. It can be seen that all these NM-GeC systems exhibit high thermodynamic stability. The most stable adsorption positions differ with NM adatoms. The F and Cl atoms prefer to adsorb at site T_Ge_, the H and P atoms prefer site T_C_ and the B, C, N, O and S atoms prefer site T_H_. The adsorption heights (D) were 1.11 Å (H), 1.11 Å (B), 0.54 Å (C), 0.91 Å (N), 1.19 Å (O), 1.82 Å (F), 1.62 Å (P), 1.81 Å (S) and 2.26 Å (Cl). For the NM-GeC systems with the same adsorption position, the lower that the adsorption energy was, the shorter that the adsorption height was and the stronger that the interaction between the adatom and the substrate atoms was. In B-, C-, N-, O-, F-, S- and Cl-GeC, the planar structure of 2D GeC was deformed for these strong interactions. Especially in C-GeC, the substrate C atom was sucked out of the GeC plane. Similar adatom-induced reconstructions of the host material were found in the NM-GaN monolayer [51].

Charge transfer occurs between the NM adatom and the substrate atoms. The NM-GeC systems adsorbed at the same position were similar in their distributions of CDD. The contributions of the same substrate atoms were equal or equidistant to the adsorbed atoms. The amount of charge transferred was determined by the relative electronegativity between the adatoms and the substrate atoms. The detailed amount of charge transferred from the adatom to the GeC was calculated by Bader charge [52,53], as +0.071|e| (H), +0.674|e| (B), −0.633|e| (C), −1.249|e| (N), −0.955|e| (O), −0.713|e| (F), −0.316|e| (P), −0.400|e| (S) and −0.555|e| (Cl). Positive values indicate charge transfer from the adatoms to the GeC, whereas negative values imply charge transfer from the GeC to the adatoms. In all the NM-GeC, only H and B atoms acted as charge donors, whereas other NM atoms acted as charge acceptors. The F and Cl atoms with stronger electronegativity had a greater influence on the substrate atoms, whose charge transfer occurred not only with the atoms near the adatoms, but also with the atoms farther away. For the NM atoms adsorbed at T_Ge_, the F atoms attracted more charge than the Cl atoms due to stronger electronegativity.

**Figure 3:**
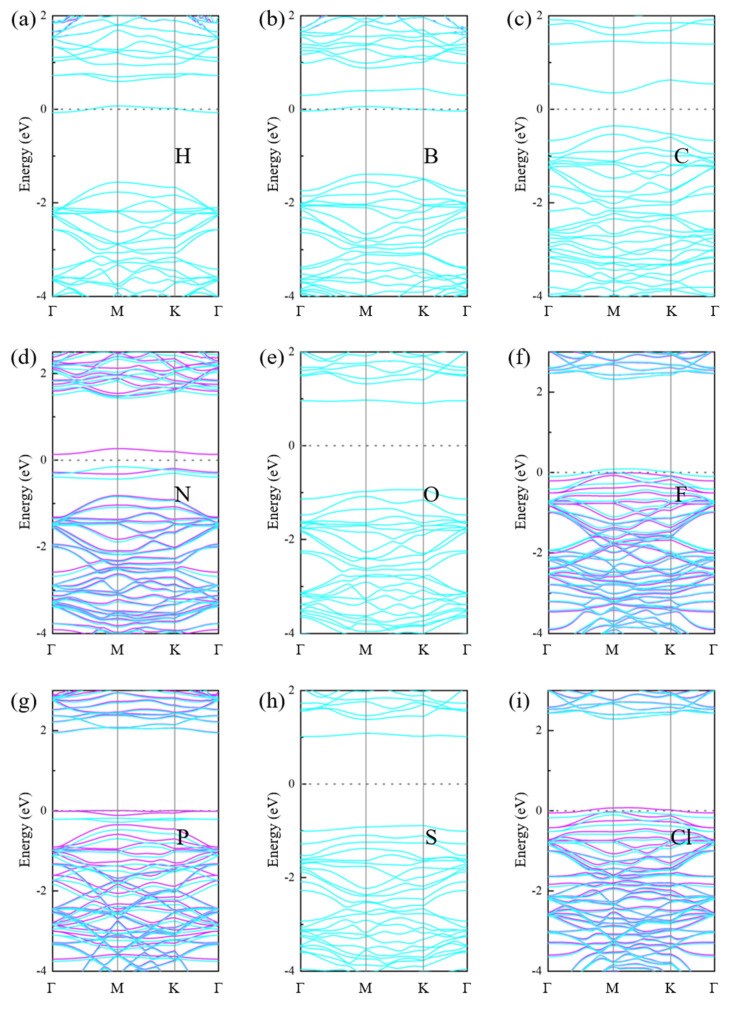
The band structures of the non-metal atoms adsorbed into GeC systems: (**a**) H-GeC, (**b**) B-GeC, (**c**) C-GeC, (**d**) N-GeC, (**e**) O-GeC, (**f**) F-GeC, (**g**) P-GeC, (**h**) S-GeC, (**i**) Cl-GeC. The blue and purple lines indicate the spin-up and the spin-down components of the energy levels. The Fermi level is shifted to zero.

**Figure 4:**
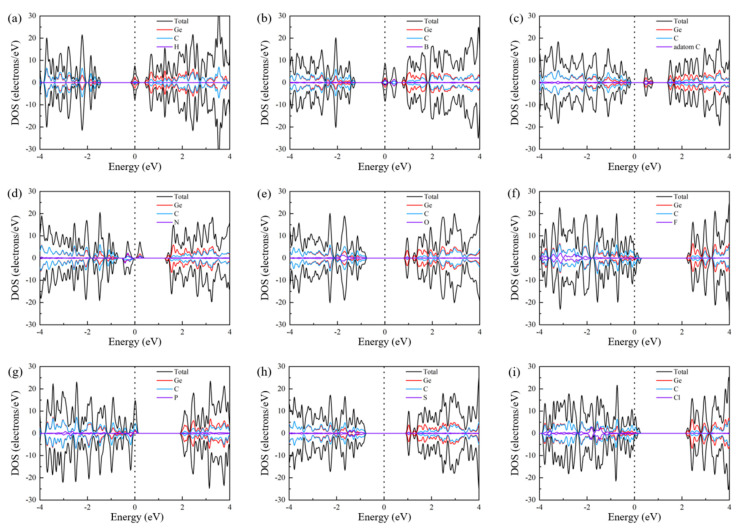
Density of states of (**a**) H-GeC, (**b**) B-GeC, (**c**) C-GeC, (**d**) N-GeC, (**e**) O-GeC, (**f**) F-GeC, (**g**) P-GeC, (**h**) S-GeC and (**i**) Cl-GeC. The spin-up component is above the Brillouin region, the spin-down component is below the Brillouin region and the Fermi level is set to zero and is represented by a vertical black dotted line.

The energy band structures and the DOS of the NM-GeC systems are shown in Figure 3 and Figure 4, respectively. It can be seen that the electronic states of the spin-up and spin-down exhibit asymmetry in N-, F-, P- and Cl-GeC, suggesting that the corresponding GeC systems were magnetic. P-GeC exhibited half-metallic properties, in which the spin-down component was metallic and the spin-up component was semiconducting. F- and Cl-GeC became magnetic metals, whose spin-up and spin-down components were asymmetric and both metallic. H- and B-GeC turned into non-magnetic metals. The C-, N-, O-, and S-GeC systems were still semiconductors, with band gaps of 0.71 eV (C), 0.28 eV (N), 1.83 eV (O) and 1.90 eV (S). Only the N-GeC system showed magnetism.

**Figure 5:**
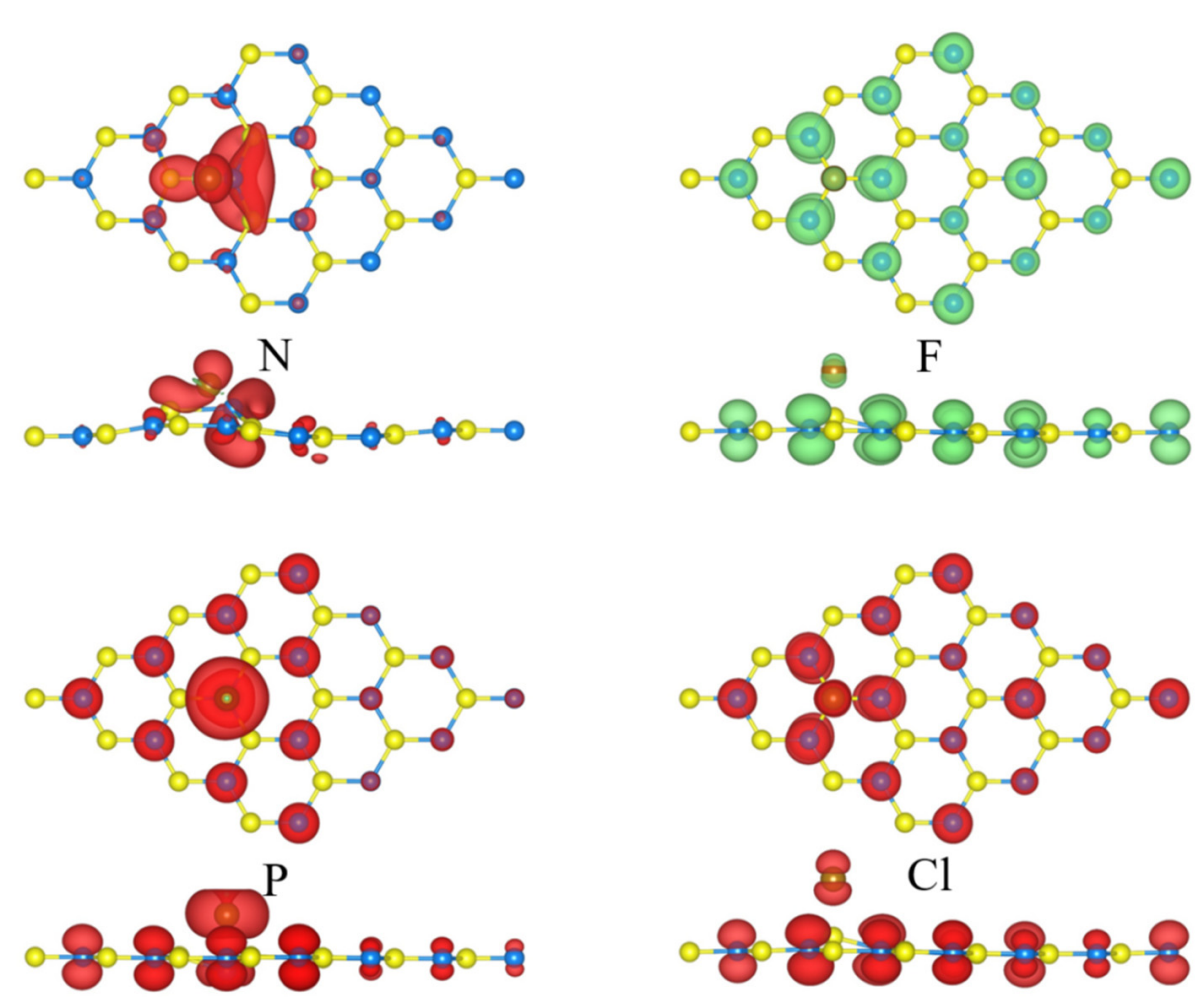
The spin-polarized charge densities of the non-metal atoms adsorbed into the GeC system. The green and red areas represent the contribution of the spin-up and spin-down charges, respectively. The isovalue is set to 0.001 e/ Å^3^.

The distribution of the spin-polarized charge densities (SPCDs) were further analyzed in the N-, F-, P- and Cl-GeC systems, as depicted in Figure 5. It can be seen that the distribution of the SPCD also shows similarities associated with the adsorption position. The same adatoms at equal distances made the same contributions to system magnetism, and the contributions of the substrate atoms should not be neglected. The distribution of the spin-polarized charge densities was extended into a large space, not just into the substrate atoms nearby, but also into those farther away, suggesting a long-range magnetic coupling interaction. This long-range magnetization is similar to that in the NM doped SiC [54].

The quantitative calculation of the magnetic moment was performed by the SPCD, and it is listed in Table 1. For the metallic NM-GeC, the corresponding magnetic moments were 0 μ_B_ (H), 0 μ_B_ (B), 0.55 μ_B_ (F) and 0.55 μ_B_ (Cl). For the semiconducting NM-GeC, they were 0 μ_B_ (C), 1 μ_B_ (N), 0 μ_B_ (O), 1 μ_B_ (P) and 0 μ_B_ (S). It can be seen that the adatoms with similar SPCD distributions responded to the same magnetic moment, such as N and P; O and S; and F and Cl.

The magnetism in the semiconducting NM-GeC can be explained by the orbital hybridization of NM atoms [39]. In the C-, N- and P-GeC systems, there occurred an *sp*^3^ orbital hybridization in the NM atoms, forming four orbits with similar energies. The valence electrons of NM atoms occupied the four orbits with the same spinning direction according to Hund’s rule and then formed four covalent bonds with the nearest IV group substrate atoms. The spinning state of the unbonded electr\ons of the NM atom determines the magnetism of NM-GeC. The remaining spinning electrons were 0 (C), 1 (N) and 1 (P), and the corresponding magnetic moments were 0 μ_B_ (C), 1 μ_B_ (N) and 1 μ_B_ (P). In the O-, and S-GeC systems, only two covalent bonds were constructed between the NM atoms and the nearest substrate atoms. The corresponding NM-GeC systems exhibited non-magnetic behavior. Although the number of covalent bonds was different, the formation of covalence bonds between the NM atom and the neighboring atoms was identical to construct more stable structures. Magnetism induced by NM adatoms expanded the application of 2D GeC in nano-spintronics devices.

**Figure 6:**
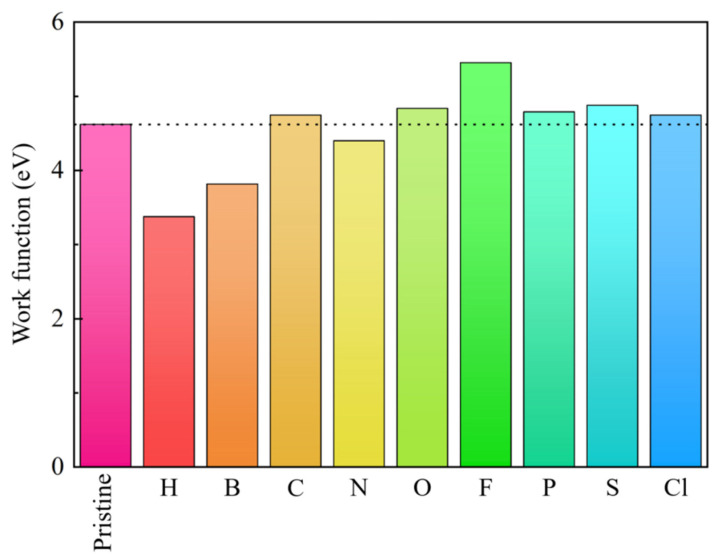
The work function of the intrinsic GeC and the NM-GeC systems.

The work function of the pristine GeC and the NM-GeC are depicted in Figure 6. The work function of GeC is 4.26 eV, which is similar to the value of conventional 2D field electron emission devices [55,56,57,58]. With the adsorption of NM atoms, the work functions of H-, B- and N-GeC were decreased, reaching a minimum of 3.37 eV in H-GeC. This indicates that less energy was required for electrons to escape to the vacuum level in NM-GeC. The corresponding electron-emitting capacity was enhanced by the adsorption of NM atoms.

**Figure 7:**
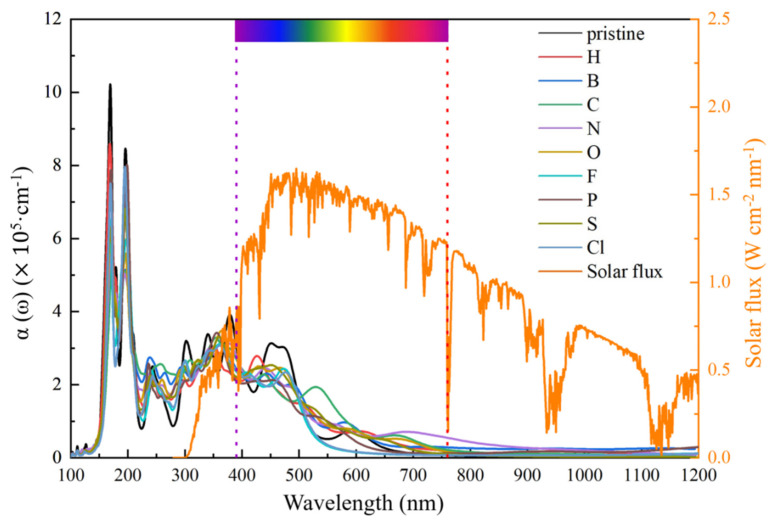
The absorption spectra of the pristine GeC and the NM-GeC systems.

The optical spectra of the pristine GeC and the NM-GeC were compared with the solar spectrum [59], as shown in Figure 7. The absorption spectrum of the pristine GeC extended from the ultraviolet to the visible light regions. The strongest absorption peak was located at a wavelength of 120 nm, with an absorption coefficient of 1.03 × 10^6^ cm^−1^, and strong visible light absorption occurred in the range of 400 to 600 nm. In the ultraviolet light region, the absorption coefficients of NM-GeC decreased. In the visible light region, a blueshift occurred in the absorption peaks in H-GeC, and a redshift occurred in that of C-GeC, with a strong absorption peak at 530 nm. Although applications in photocatalysts are limited by the bandgap, the 2D NM-GeC shows its fantastic advantages as a photovoltaic cell and ultraviolet photoelectric detector.

## 4. Conclusions

Nine NM-atoms adsorbed into GeC systems were constructed based on the principle of favorable thermodynamics. The most stable adsorption positions were distinguished with NM atoms. F and Cl atoms were more likely to absorb at site T_Ge_, H and P atoms preferred site T_C_, and the B, C, N, O and S atoms preferred site T_H_. C-GeC exhibited the strongest adsorption and the shortest adsorption height. The adsorption of NM atoms induced variations in the properties of NM-GeC. F- and Cl-GeC became magnetism metals, P-GeC turned into a half-metal and H- and B-GeC systems exhibited the properties of non-magnetic metals. C- and O-GeC were non-magnetic semiconductors, whereas N-GeC appeared as a magnetic semiconductor. The work function decreased in the H-, B- and N-SiC systems and achieved a minimum in the H-GeC system. The absorption spectrum of C-GeC redshifted in the visible light region and had a strong absorption peak at 530 nm. Magnetism in semiconducting NM-GeC is explained by the spinning states of the unbonded electrons of the NM atoms. After the covalence electrons of NM atoms formed covalent bonds with adjacent IV group atoms, the remaining spinning electrons determined the magnetism of the NM-GeC system. These electrical, magnetic and optical properties caused by the adsorption of non-metal atoms extend the application of 2D GeC, especially in field electron-emitting, spin electronics, photovoltaic cells and ultraviolet photoelectric detectors. Our discussion on the magnetism of semiconducting NM-GeC provides a reference for other NM 2D materials.

## Figures and Tables

**Table 1:** The adsorption energy (*E*_ad_), charge transferred from the adatom to the GeC substrate (*C*), magnetic moment (*M*_total_), bandgap (*E*_g_) and the adsorption height (d) of the non-metal atoms adsorbed into GeC systems.

Adsorption Atom	Adsorption Site	*E*_ad_ (eV)	*C* (e)	*M*_total_ (μ_B_)	*E*_g_ (eV)	d (Å)
H	T_C_	−3.252	+0.071	0	0	1.11
B	T_H_	−5.416	+0.674	0	0	1.11
C	T_H_	−9.343	−0.633	0	0.71	0.54
N	T_H_	−7.929	−1.249	1	0.28	0.91
O	T_H_	−5.973	−0.955	0	1.83	1.19
F	T_Ge_	−4.583	−0.713	0.55	0	1.82
P	T_C_	−4.089	−0.316	1	0	1.62
S	T_H_	−4.092	−0.400	0	1.90	1.81
Cl	T_Ge_	−2.933	−0.555	0.55	0	2.26

## Data Availability

Not applicable.
